# Impact of trauma level designation on mortality in trauma patients with sepsis: an observational study across US trauma centers

**DOI:** 10.3389/fmed.2025.1591624

**Published:** 2025-08-18

**Authors:** Ralphe Bou Chebl, Razan Diab, Reem Siblini, Rana Bachir, Mazen El Sayed

**Affiliations:** Department of Emergency Medicine, American University of Beirut Medical Center, Beirut, Lebanon

**Keywords:** trauma, sepsis, trauma center levels, mortality, National Trauma Data Bank

## Abstract

**Background:**

Sepsis is a major complication in trauma patients, leading to increased morbidity and mortality. Given the varying resource allocation across trauma center levels, the impact of trauma center designation on sepsis-related mortality remains unclear. This study examines the association between trauma center level and sepsis outcomes in trauma patients using data from the National Trauma Data Bank (NTDB) 2017 dataset.

**Methods:**

A retrospective cohort study was conducted using the NTDB 2017 dataset at the American University of Beirut (AUB). Trauma patients who developed sepsis as a hospital complication were identified, and those meeting inclusion criteria were analyzed. Patient demographics, comorbidities, injury severity, hospital characteristics, and outcomes were compared across Level I, II, and III trauma centers. Multivariable logistic regression was performed to assess the association between trauma center designation and mortality after adjusting for confounders.

**Results:**

A total of 1,738 patients were included. The study population had a mean age of 56.34 ± 19.54 years, with 72.9% being males and 69.2% of white race. Patients treated in a level I trauma center had a higher injury severity score (ISS ≥ 16) compared to those in other trauma center levels (62.9% vs. 54.5% vs. 22.6%, *p* < 0.001), and increased hospital complications, including ventilator-associated pneumonia (20% vs. 10.7% vs. 5.2%, *p* < 0.001). ICU and OR admissions were significantly higher in Level I and II trauma centers than in Level III (47.9% and 45.9% vs. 30.4% and 30.9%, and 24.1% vs. 13%, *p* < 0.001). Mortality rates were highest in Level I centers (62.4%) compared to Level II (30.8%) and Level III (6.8%), though this difference was not statistically significant after adjustment for confounders (*p* = 0.691). Multivariable analysis showed no significant association between trauma center designation and sepsis-related mortality when comparing Level II to Level I centers (OR = 0.785, 95% CI: 0.592–1.043; *p* = 0.095) and Level III to Level I centers (OR = 1.038, 95% CI: 0.454–2.372; *p* = 0.930).

**Conclusion:**

Sepsis-related mortality did not significantly differ across trauma level designation when adjusted for potential confounders. These findings highlight the importance of standardized sepsis management protocols across trauma centers as well as the importance of early sepsis recognition and intervention strategies in trauma patients.

## 1 Introduction

Trauma centers are designated and verified by the American College of Surgeons (ACS) into different levels (I, II, III, IV, or V) with state-dependent variations. This division is based on the availability of resources, trauma volume, and commitment to education and research (1). Proper trauma system organization and triage has led to improved trauma outcomes and reduced mortality rates, particularly in severe traumatic injuries (2–7). Prior studies evaluating patient outcomes based on trauma center levels showed Level I trauma centers to have higher survival rates for patients with severe injuries (Injury Severity Score ISS > 15 and > 25) compared to other levels (8–14). As level I trauma centers are equipped with advanced surgical and critical care capabilities, this allows for timely interventions during the “golden hour”—the critical 60 min window following a trauma (15). Despite advances in trauma designation, trauma remains a leading cause of death worldwide, particularly in the fourth decade of life, with 60% of mortality occurring within the initial hours of hospital admission (15, 16). This is believed to be largely due to secondary complications such as sepsis, one of the most frequent and life-threatening sequelae of trauma hospitalization (16, 17). It is estimated that 10% of trauma patients develop sepsis within the first 4 days of admission (16). In the context of trauma, mechanical disruption of protective barriers and exposure to exogenous pathogens —often introduced during diagnostic and therapeutic interventions— can significantly increase the risk of infection and subsequent sepsis (16). Beyond its impact on morbidity and mortality, sepsis also results in more ICU admissions, increased healthcare costs, worse post-trauma functional disability, and prolonged hospital stays (16). Therefore, early recognition of sepsis and timely initiation of resuscitation are critical to improving outcomes and reducing complications such as septic shock, ICU admission, and death. Although trauma center designation has been linked to improved survival in patients with severe injuries, its specific effect on outcomes in trauma patients who develop sepsis remains an underexplored gap in the literature. One study showed that trauma service implementation in surgical ICUs is associated with decreased mortality, primarily due to reductions in sepsis and multi-organ failure (6). These findings suggest that trauma center designation and effective resource allocation may play a critical role not only in initial trauma care but also in mitigating downstream complications. We hypothesize that trauma patients who develop sepsis are more likely to experience better outcomes, including lower in-hospital mortality, when treated at Level I trauma centers. Level I trauma centers typically possess more advanced resources compared to other levels, including greater ICU capabilities, specialized staff and established protocols that allows for early sepsis identification and management (12).

Therefore, the aim of this study is to investigate whether trauma center level designation, reflecting differences in resource availability and critical care infrastructure, influences outcomes in trauma patients who develop sepsis. Addressing this gap may help optimize resource allocation and adequate cost distribution across trauma centers as well as enhance sepsis recognition strategies and management to improve trauma patient outcomes and reduce sepsis-related mortality.

## 2 Materials and methods

### 2.1 Study approval

This study was approved by the Institutional Review Board (IRB) of the American University of Beirut (AUB). Since the National Trauma Data Bank (NTDB) data is completely anonymous, IRB approval was originally granted for all research studies utilizing it. Informed consent was waived given its retrospective nature and the use of NTDB de-identified data. Additionally, this study did not entail any direct contact or involvement in the clinical care of participants, and waiving consent did not adversely affect their welfare.

### 2.2 Study design

This retrospective cohort study included all trauma patients above 15 years who developed sepsis and were registered in the National Trauma Data Bank (NTDB; American College of Surgeons; Chicago, IL, United States) 2017 dataset. NTDB is considered the largest trauma registry in the United States, gathering data from more than 900 trauma facilities and releasing its datasets annually (18). Patient inclusion in NTDB is done using International Classification of Diseases (ICD) codes for trauma related injuries. ICD codes of superficial injuries are excluded. Information collected includes pre-hospital, emergency department (ED), and hospital data, including patient demographics, injury details, diagnoses, procedures, dispositions, and outcomes (19). We chose the 2017 dataset because we had bought earlier access for previous projects. Trauma designation level for the centers was selected as the highest level given by either the ACS designation or the State designation (20). A Level I trauma center was defined as a university-based teaching hospital equipped with system leadership and extensive resources to provide acute care for all trauma injuries, expand capacity in education and regional disaster planning and advance trauma care research. Level II trauma centers; on the other hand, provide adequate trauma care for most injuries of different severities and can potentially contribute to education, system leadership, and disaster planning. Finally, level III trauma centers are dedicated to serve rural communities with limited access to Level I or II trauma centers, managing mild to moderate injuries and ensuring prompt evaluation, initial management, and transfer of patients with more serious injuries exceeding the center’s available resources (21).

### 2.3 Study sample selection and outcome

**FIGURE 1:**
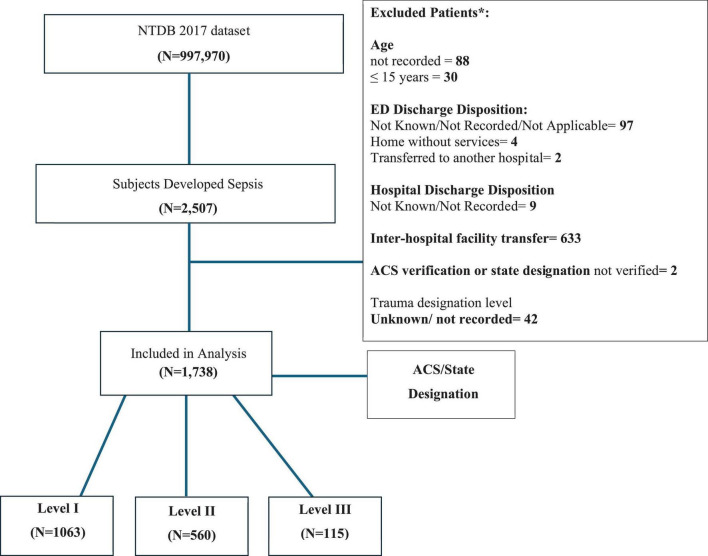
Flow diagram. The diagram is a visual summary illustrating the selection methodology of the study sample from the National Trauma Data Bank (NTDB) database. Stage 1 shows total number of trauma patients available in the 2017 dataset. Stage 2 represents number of trauma patients who developed sepsis as a hospital complication. Stage 3 shows final number included in analysis after inclusion/exclusion criteria. Stage 4 represents sub-classification of subjects into the three levels of trauma designation. Numbers represent count of patient in each group. *There are overlaps among the categories of the excluded variables which explain why the final number of data included in analysis cannot be calculated by subtracting the sum of patients pertaining to each exclusion criterion from the selected NTDB data.

All patients older than 15 years who were admitted for a trauma injury and developed sepsis as a complication were included. Sepsis was defined according to sepsis-3 definition (22). NTDB data dictionary stated a diagnosis of sepsis must be documented in the patient’s medical record and must have occurred during the patient’s initial stay at the hospital with symptom onset after arrival to the ED/hospital (19). The definitions of sepsis variables reported are consistent with the American College of Chest Physicians and the Society of Critical Care (19). Patients ≤ 15 years or whose age was not recorded were excluded. Additional exclusion criteria were emergency department (ED) discharge disposition not known/not recorded/not applicable, discharged home without services, transferred to another hospital, hospital discharge disposition not known/not recorded, inter-hospital facility transfer, patients who presented to ACS verification level as “not verified” or state designation as “not verified” and patients with unknown/not recorded trauma designation level. Included patients were then classified into three trauma levels according to the ACS/state designation: 1,063 patients in Trauma level I, 560 patients in Trauma level II, and 115 patients in Trauma level III (Figure 1).

Our data was extracted from NTDB –the largest and the most representative trauma database in the United States. To avoid the occurrence of selection bias and false negative results (type II error), all eligible patients were pulled out from the database. Multiple studies reported the validity of NTDB and the accuracy of data abstraction, demonstrating the precision of context-specific generalizability of study findings generated using NTDB data (23, 24).

The primary outcome selected was mortality among trauma patients who developed sepsis across the three levels of trauma center designation.

### 2.4 Statistical analysis

Data analysis was done using Statistical Package for Social Sciences (SPSS), version 27.0 (IBM, Armonk, NY, United States). Categorical variables were described by frequencies and percentages, while age was summarized by presenting the mean and standard deviation (SD). All categorical factors were tabulated by the main independent variable (trauma designation level) and the study outcome (died: yes/no) and compared using the Pearson’s Chi-Square or the Fisher’s exact tests. A logistic regression using a stepwise selection procedure was conducted to calculate the odds ratios (ORs) with the 95% confidence intervals (95% CIs) and to evaluate the association of mortality with the trauma level designation in trauma patients who developed sepsis. Notably, all clinically and/or statistically significant factors were included in the regression analysis. The following independent variables were adjusted for in this model: age (years), sex, race, ethnicity, primary method of payment, transport mode, hospital teaching status, hospital type, bed size, trauma designation level, comorbidity, Injury Severity score (ISS), Glasgow Coma Scale (GCS), Systolic Blood Pressure (SBP), blood transfusion (in 4 h), trauma type, injury intentionality, mechanism of Injury, alcohol screen, drug screen, nature of injury, body region, signs of life and hospital complications [Central Line-Associated Bloodstream Infection (CLABSI); Deep Surgical Site Infection; Alcohol Withdrawal Syndrome; Cardiac Arrest with Cardiopulmonary Resuscitation (CPR); Catheter-Associated Urinary Tract Infection (CAUTI); Pulmonary Embolism (PE); Extremity Compartment Syndrome; Unplanned Intubation; Acute Kidney Injury (AKI); Myocardial Infarction (MI); Organ/Space Surgical Site Infection; Osteomyelitis; Acute Respiratory Distress Syndrome (ARDS); Unplanned Return to the OR; Stroke/Cerebrovascular Accident (CVA); Superficial Incisional Surgical Site Infection; Unplanned Admission to the ICU; Other]. Crude and adjusted ORs of patient mortality were compared across the three trauma designation levels and statistical significance was set at a *p*-value of ≤ 0.05. The Omnibus Tests of Model Coefficients revealed that after controlling for all confounding factors, the final model is an improvement over the baseline model that contains only the intercept (*p* < 0.001). The Hosmer-Lemeshow goodness of fit test revealed that the final model is a good fit to the data (*p* = 0.087). In addition, there are no influential observations, as the Cook Distance of all cases was less than one. All values of the variance inflation factors of the independent factors were less than 10 and this indicated the absence of a multicollinearity problem in the regression model.

## 3 Results

The total number of trauma patients included in the NTDB 2017 dataset was 997,970. Of those, 2,507 developed sepsis as a hospital complication. After excluding patients based on age (118), ED discharge disposition (103), hospital discharge disposition (9), inter-hospital facility transfer (633), and ACS verification and/or state designation (44), 1,738 patients were included for analysis.

The mean age of the total sample was 56.34 years (SD = 19.54 years) with 35.8% of patients being older than 65. 72.9% of them being males and 69.2% were of Caucasian race. The majority of patients across all three trauma levels were older than 66 years, with the highest proportion observed in Level III (31.6% vs. 39.3% vs. 57.4, *p* < 0.001). Additionally, sex and race distribution varied significantly across the three trauma levels with Caucasian males comprising the majority of the cohort at each level (*p* < 0.001). In our sample, patients treated across all three trauma centers were more likely to have comorbidities than not (*p* = 0.002). Among the commonly reported comorbidities in NTDB are diabetes mellitus (DM), hypertension (HTN) and chronic obstructive pulmonary disease (COPD). Most patients presenting to the emergency department post trauma had a negative alcohol screen, although a higher percentage of severe positive alcohol screen was observed in trauma Level III (19%) compared to levels I (13%) and II (7.2%) (*p* = 0.005). Drug screen was also negative in the majority of patients across all three levels, yet the variation was not statistically significant (*p* = 0.236).

The majority of patients in Level I trauma centers were treated in university hospitals (70.9%), compared to community hospitals for Level II centers (65%) and non-teaching hospitals for Level III centers (58.3%) (< 0.001). The majority of patients were brought in by ground ambulance across the three hospitals, while helicopter use was more common in Level I (16%) compared to Levels II (8.4%), and III (2.6%) (*p* < 0.001) (Table 1).

**TABLE 1:** Demographics of trauma patients and facility characteristics.

	Total	Trauma designation level			*P*-value
	*N* = 1,738	I (*N*_1_ = 1,063)	II (*N*_2_ = 560)	III (*N*_3_ = 115)	
**Age (years)**					
16–25	152 (8.7%)	98 (9.2%)	47 (8.4%)	7 (6.1%)	< 0.001
26–35	179 (10.3%)	124 (11.7%)	47 (8.4%)	8 (7.0%)	
36–45	182 (10.5%)	120 (11.3%)	59 (10.5%)	3 (2.6%)	
46–55	247 (14.2%)	165 (15.5%)	73 (13.0%)	9 (7.8%)	
55–65	356 (20.5%)	220 (20.7%)	114 (20.4%)	22 (19.1%)	
≥ 66	622 (35.8%)	336 (31.6%)	220 (39.3%)	66 (57.4%)	
**Sex**					
Male	1,267 (72.9%)	803 (75.6%)	398 (71.1%)	66 (57.4%)	< 0.001
Female	470 (27.0%)	259 (24.4%)	162 (28.9%)	49 (42.6%)	
Not known/not recorded	1 (0.1%)	–	–	–	
**Race**					
Black	302 (17.4%)	221 (21.3%)	77 (14.0%)	4 (3.5%)	< 0.001
White	1,202 (69.2%)	695 (67.1%)	412 (74.9%)	95 (82.6%)	
Other race*/not known/not recorded	234 (13.5%)	120 (11.6%)	61 (11.1%)	16 (13.9%)	
**Comorbidity**					
No	342 (19.7%)	226 (21.3%)	107 (19.1%)	9 (7.8%)	0.002
Yes	1396 (80.3%)	837 (78.7%)	453 (80.9%)	106 (92.2%)	
**Alcohol screen result**					
Negative screening	768 (70.4%)	496 (70.8%)	247 (71.0%)	25 (59.5%)	0.005*
Mild to moderate	199 (18.2%)	114 (16.3%)	76 (21.8%)	9 (21.4%)	
Severe	124 (11.4%)	91 (13.0%)	25 (7.2%)	8 (19.0%)	
**Drug screen**					
No	1,336 (76.9%)	807 (79.7%)	434 (78.5%)	95 (85.6%)	0.236
Yes	340 (19.6%)	205 (20.3%)	119 (21.5%)	16 (14.4%)	
Not known/not recorded	62 (3.6%)	–	–	–	
**Transport mode**					
Ground ambulance	1,382 (79.5%)	814 (77.2%)	466 (83.4%)	102 (88.7%)	< 0.001*
Helicopter ambulance	219 (12.6%)	169 (16.0%)	47 (8.4%)	3 (2.6%)	
Private/public vehicle/walk-in	104 (6.0%)	51 (4.8%)	44 (7.9%)	9 (7.8%)	
Police and other	23 (1.3%)	20 (1.9%)	2 (0.4%)	1 (0.9%)	
**Facility level: hospital teaching status**					
Community	686 (39.5%)	284 (26.7%)	364 (65.0%)	38 (33.0%)	< 0.001
Non-teaching	201 (11.6%)	25 (2.4%)	109 (19.5%)	67 (58.3%)	
University	851 (49.0%)	754 (70.9%)	87 (15.5%)	10 (8.7%)	
**Facility level: bed size**					
≤ 200	100 (5.8%)	46 (4.3%)	35 (6.3%)	19 (16.5%)	< 0.001
201–400	466 (26.8%)	124 (11.7%)	277 (49.5%)	65 (56.5%)	
401–600	531 (30.6%)	369 (34.7%)	134 (23.9%)	28 (24.3%)	
> 600	641 (36.9%)	524 (49.3%)	114 (20.4%)	3 (2.6%)	

*Other race is the combination of the following categories: Asian and Pacific Islander and American Indian and Other Race. N, total number of trauma patients with sepsis after exclusion; N1, total number of patients treated in level I trauma center; N2, total number of patients treated in level II trauma center; N3, total number of patients treated in level III trauma center. Most patients across all three trauma levels were above 66 years, of white race, with comorbidities and transported via ground ambulance. Patients in Level I trauma centers were mainly treated in university hospitals compared to community hospitals in Level II and non-teaching hospitals in Level III. Data extracted from the National Trauma Data Bank, 2024 United States.

Patients who were admitted to Level I and II centers had lower GCS than those treated at a Level III facility (GCS ≤ 8 22% vs. 17.2% vs. 8.2%, and GCS 9-12 7.2% vs. 8% vs. 4.5%, *p* = 0.001). On the other hand, SBP of patients upon presentation was relatively similar across the three groups, most of which measured ≥ 91 mmHg (85.5% vs. 86.7% vs. 88.4%, *p* = 0.608). In terms of blood transfusion, the highest percentage of patients who received blood within 4 h of presentation was reported in level 1 trauma center compared to others (33.1% vs. 24.8% vs. 13.6%, *p* < 0.001).

Table 2 summarizes the injury characteristics including severity score (ISS), trauma type, mechanism of injury and nature of injury as well as patients’ disposition. Patients treated in a level I trauma center had a higher severity score (ISS ≥ 16) compared to patients in other trauma centers (62.9% vs. 54.5% vs. 22.6%, *p* < 0.001). Blunt trauma was the most common trauma type across the three groups (80.9% vs. 87.3% vs. 91.2%, *p*-value < 0.001). In terms of mechanism of injury, trauma patients in level I centers were more likely to have suffered from a motor vehicle trauma (MVT) compared to falls (39.1% and 33.7%, respectively), while falls were the most encountered injury mechanism in trauma levels II and III (44.4% and 68.1%, respectively) (*p* < 0.001). The two most common injuries among patients in all three trauma designation levels were internal organ injury or a fracture (40.7% vs. 38.8% vs. 25.4% for and 35.7% vs. 43.6% vs. 50%, respectively) (*p* < 0.001). The remaining injuries were similar among the three groups, and findings were statistically significant for all types.

**TABLE 2:** Parameters of emergency department (ED) presentation, treatment and disposition among patients in trauma designation levels I, II, and III.

	Total	Trauma designation level			*P*-value
	*N* = 1,738	I (*N*_1_ = 1,063)	II (*N*_2_ = 560)	III (*N*_3_ = 115)	
**GCS**					
Severe ≤ 8	325 (18.7%)	226 (22.0%)	90 (17.2%)	9 (8.2%)	0.001
Moderate 9–12	121 (7.0%)	74 (7.2%)	42 (8.0%)	5 (4.5%)	
Mild 13–15	1,213 (69.8%)	725 (70.7%)	392 (74.8%)	96 (87.3%)	
Not known/not recorded	79 (4.5%)	–	–	–	
**SBP**					
≤ 90	236 (13.6%)	151 (14.5%)	72 (13.3%)	13 (11.6%)	0.608
≥ 91	1,460 (84.0%)	890 (85.5%)	471 (86.7%)	99 (88.4%)	
Not known/not recorded	42 (2.4%)	–	–	–	
**Transfusion blood (4 h)**					
No	1,148 (66.1%)	704 (66.9%)	406 (75.2%)	38 (86.4%)	< 0.001
Yes	488 (28.1%)	348 (33.1%)	134 (24.8%)	6 (13.6%)	
Not known/not recorded	102 (5.9%)	–	–	–	
**ISS**					
≤ 15	737 (42.4%)	393 (37.1%)	255 (45.5%)	89 (77.4%)	< 0.001
≥ 16	998 (57.4%)	667 (62.9%)	305 (54.5%)	26 (22.6%)	
Not known/not recorded	3 (0.2%)	–	–	–	
**Trauma type**					
Blunt	1,445 (83.1%)	854 (80.9%)	487 (87.3%)	104 (91.2%)	< 0.001*
Penetrating	215 (12.4%)	142 (13.5%)	66 (11.8%)	7 (6.1%)	
Burn	38 (2.2%)	37 (3.5%)	1 (0.2%)	0 (0%)	
Other/unspecified/not known/not recorded	40 (2.3%)	22 (2.1%)	4 (0.7%)	3 (2.6%)	
**Mechanism of injury**					
Fall	677 (39.0%)	354 (33.7%)	246 (44.4%)	77 (68.1%)	< 0.001
Firearm	176 (10.1%)	114 (10.9%)	56 (10.1%)	6 (5.3%)	
MVT	622 (35.8%)	411 (39.1%)	191 (34.5%)	20 (17.7%)	
Other*	242 (13.9%)	171 (16.3%)	61 (11.0%)	10 (8.8%)	
Not known/not recorded	21 (1.2%)	–	–	–	
**Nature of injury**					
Fracture	677 (39.0%)	376 (35.7%)	244 (43.6%)	57 (50.0%)	< 0.001
Internal organ injury	675 (38.8%)	429 (40.7%)	217 (38.8%)	29 (25.4%)	
Open wound	125 (7.2%)	77 (7.3%)	77 (7.3%)	12 (10.5%)	
Superficial and contusion	98 (5.6%)	60 (5.7%)	29 (5.2%)	9 (7.9%)	
Other**/not known/not recorded	163 (9.4%)	111 (10.5%)	34 (6.1%)	7 (6.1%)	
**ED discharge disposition**					
Floor bed (general admission, non-specialty unit bed)	335 (19.3%)	158 (14.9%)	128 (22.9%)	49 (42.6%)	< 0.001
Observation unit (unit that provides < 24 h stays)	18 (1.0%)	14 (1.3%)	1 (0.2%)	3 (2.6%)	
Telemetry/step-down unit (less acuity than ICU)	103 (5.9%)	52 (4.9%)	39 (7.0%)	12 (10.4%)	
Operating room	478 (27.5%)	328 (30.9%)	135 (24.1%)	15 (13.0%)	
Intensive Care Unit (ICU)	801 (46.1%)	509 (47.9%)	257 (45.9%)	35 (30.4%)	
Home without services	3 (0.2%)	2 (0.2%)	0 (0%)	1 (0.9%)	
**Hospital discharge disposition**					
Deceased/expired	558 (32.1%)	348 (32.7%)	172 (30.7%)	38 (33.0%)	0.391*
Left against medical advice or discontinued care	11 (0.6%)	10 (0.9%)	1 (0.2%)	0 (0%)	
Discharged to home or self-care (routine discharge)	174 (10.0%)	104 (9.8%)	58 (10.4%)	12 (10.4%)	
Transferred to other destination	992 (57.1%)	599 (56.3%)	329 (58.8%)	64 (55.7%)	
Not applicable	3 (0.2%)	2 (0.2%)	0 (0%)	1 (0.9%)	

*Other mechanism of injury includes: Cut/pierce and Fire/flame and Hot object/substance and Machinery and Pedal cyclist, other and Pedestrian, other and Transport, other and Natural/environmental, Bites and stings and Natural/environmental, Other and Overexertion and Poisoning and Struck by, against and Other specified and classifiable and Other specified, not elsewhere classifiable and Unspecified. **Other nature of injury includes: Amputation and Blood vessel and Burns and corrosions and Crushing and Dislocation and Other effects of external causes and Other specified injury and Toxic effects and Unspecified injury. N, total number of trauma patients with sepsis after exclusion; N1, total number of patients treated in level I trauma center; N2, total number of patients treated in level II trauma center; N3, total number of patients treated in level III trauma center. Table 2 summarizes the different injury characteristics, treatment parameters and discharge disposition among trauma patients in the three trauma designation levels. Significant associations were reported between trauma designation level and each of Glascow Coma Scale, blood transfusion at 4 h, injury severity score, trauma type, mechanism and nature of injury and ED discharge disposition. Data extracted from the National Trauma Data Bank, 2024 United States.

Intensive Care Unit and OR admissions were significantly higher in Level I and II trauma centers than in Level III (47.9% and 45.9% vs. 30.4% and 30.9%, and 24.1% vs. 13%, *p* < 0.001). In contrast, Level III had the highest general admission rate to non-specialty floor beds (42.6%) (Table 2).

In terms of hospital complications, patients treated in level I trauma center were more likely to develop DVT (11.6% vs. 10.5% vs. 3.5%, *p* = 0.028), pressure ulcers (11.3% vs. 5.7% vs. 0.9%, *p* < 0.001) and VAP (20% vs. 10.7% vs. 5.2%, *p* < 0.001) and experience an unplanned return to the OR (13.2% vs. 9.5% vs. 6.1%, *p* = 0.014) in comparison to levels II and III. However, no statistical significance was reported among the three trauma designation levels for the other hospital complications reported in Table 3.

**TABLE 3:** Hospital complications in trauma patients with sepsis across the three trauma level designations.

	Total	Trauma designation level			*P*-value
	*N* = 1,738	I (*N*_1_ = 1,063)	II (*N*_2_ = 560)	III (*N*_3_ = 115)	
Central line-associated bloodstream infection (CLABSI)	40 (2.3%)	27 (2.5%)	11 (2.0%)	2 (1.7%)	0.808*
Deep surgical site infection	62 (3.6%)	47 (4.4%)	13 (2.3%)	2 (1.7%)	0.067*
Deep vein thrombosis (DVT)	186 (10.7%)	123 (11.6%)	59 (10.5%)	4 (3.5%)	0.028
Cardiac arrest with CPR	203 (11.7%)	123 (11.6%)	70 (12.5%)	10 (8.7%)	0.504
Catheter-associated urinary tract infection (CAUTI)	88 (5.1%)	63 (5.9%)	22 (3.9%)	3 (2.6%)	0.101
Extremity compartment syndrome	19 (1.1%)	13 (1.2%)	5 (0.9%)	1 (0.9%)	0.863*
Unplanned intubation	427 (24.6%)	267 (25.1%)	124 (22.1%)	36 (31.3%)	0.092
Acute kidney injury	437 (25.1%)	265 (24.9%)	148 (26.4%)	24 (20.9%)	0.442
Organ/space surgical site infection	57 (3.3%)	34 (3.2%)	21 (3.8%)	2 (1.7%)	0.588*
Osteomyelitis	6 (0.3%)	6 (0.6%)	0 (0%)	0 (0%)	0.219*
Acute respiratory distress syndrome (ARDS)	289 (16.6%)	165 (15.5%)	104 (18.6%)	20 (17.4%)	0.285
Unplanned return to the OR	200 (11.5%)	140 (13.2%)	53 (9.5%)	7 (6.1%)	0.014
Superficial incisional surgical site infection	36 (2.1%)	23 (2.2%)	13 (2.3%)	0 (0%)	0.291*
Pressure ulcer	153 (8.8%)	120 (11.3%)	32 (5.7%)	1 (0.9%)	< 0.001
Ventilator-associated pneumonia (VAP)	279 (16.1%)	213 (20.0%)	60 (10.7%)	6 (5.2%)	< 0.001

*Indicates that the Fisher’s exact was used to calculate the *p*-value. N, total number of trauma patients with sepsis after exclusion; N1, total number of patients treated in level I trauma center; N2, total number of patients treated in level II trauma center; N3, total number of patients treated in level III trauma center. Patients treated in level I trauma center were more likely to develop DVT, pressure ulcers and VAP and experience an unplanned return to the OR. Other hospital complications showed no statistical significance among the three trauma designation levels. Data extracted from the National Trauma Data Bank, 2024 United States.

Mortality was higher (62.4%) in level I trauma centers compared to 30.8% in level II, and 6.8% in level III, but this finding was not significant (*p*-value = 0.691). The highest mortality was observed in patients ≥ 66 years (*p*-value < 0.001) and those with a negative alcohol screen (*p*-value = 0.026). On the other hand, mortality did not vary with respect to patient sex, race, comorbidity, transport mode, hospital teaching status and bed size (0.203 - 0.171 - 0.205 - 0.405 - 0.563 - 0.449, respectively) (Table 4.1). Moreover, significant mortality was documented for patients with a mild GCS score 13–15 (69%, *p*-value = 0.029) and SBP ≥ 91 (82.5%, *p*-value = 0.003). Blunt trauma (85.8%) was more likely to cause death than other trauma types (*p*-value < 0.001). Similarly, falls (42.9%) and MVT (36.5%) resulted in higher mortality rates than firearms (7.1%) and other mechanisms of injury (13.5%) (*p*-value = 0.016) (Table 4.2). After adjusting for clinically and statistically significant variables, including patient demographics, injury details (mechanism, type and ISS), mode of transportation, complications and hospital teaching status and details, there was no statistical difference in mortality of trauma patients with sepsis between those taken to Level I and II centers (OR = 0.785, 95% CI: 0.592–1.043; *p* = 0.095) and Level I and III centers (OR = 1.038, 95% CI: 0.454–2.372; *p* = 0.930) (Table 5).

**TABLE 4.1:** Mortality in sample of trauma patients from National Trauma Data Bank (NTDB) with respect to patient demographics and facility characteristics.

	Died		*P*-value
	No (*N* = 1,180)	Yes (*N* = 558)	
**Trauma designation level**			
I	715 (60.6%)	348 (62.4%)	0.691
II	388 (32.9%)	172 (30.8%)	
III	77 (6.5%)	38 (6.8%)	
**Age (years)**			
16–25	122 (10.3%)	30 (5.4%)	< 0.001
26–35	145 (12.3%)	34 (6.1%)	
36–45	141 (11.9%)	41 (7.3%)	
46–55	186 (15.8%)	61 (10.9%)	
56–65	220 (18.6%)	136 (24.4%)	
≥ 66	366 (31.0%)	256 (45.9%)	
**Sex**			
Male	871 (73.9%)	396 (71.0%)	0.203
Female	308 (26.1%)	162 (29.0%)	
**Race**			
Black	213 (18.4%)	89 (16.3%)	0.171
White	800 (69.3%)	402 (73.6%)	
Other race*	142 (12.3%)	55 (10.1%)	
**Comorbidity**			
No	242 (20.5%)	100 (17.9%)	0.205
Yes	938 (79.5%)	458 (82.1%)	
**Alcohol screen result**			
Negative screening	513 (67.9%)	255 (75.9%)	0.026
Mild to moderate	151 (20.0%)	48 (14.3%)	
Severe	91 (12.1%)	33 (9.8%)	
**Transport mode**			
Ground ambulance	925 (78.9%)	457 (82.3%)	0.405
Helicopter ambulance	157 (13.4%)	62 (11.2%)	
Private/public vehicle/walk-in	75 (6.4%)	29 (5.2%)	
Police and other	16 (1.4%)	7 (1.3%)	
**Facility level: hospital teaching status**			
Community	475 (40.3%)	211 (37.8%)	0.563
Non-teaching	132 (11.2%)	69 (12.4%)	
University	573 (48.6%)	278 (49.8%)	
**Facility level: bed size**			
≤ 200	70 (5.9%)	30 (5.4%)	0.449
201–400	322 (27.3%)	144 (25.8%)	
401–600	368 (31.2%)	163 (29.2%)	
> 600	420 (35.6%)	221 (39.6%)	

*Other race is the combination of the following categories: Asian and Pacific Islander and American Indian and Other Race. N, number of patients in each group. Table 4.1 summarizes associations of mortality with trauma designation levels, patient demographics and facility characteristics. Mortality was highest in trauma level I but findings were not statistically significant. Data extracted from the National Trauma Data Bank, 2024 United States.

**TABLE 4.2:** Mortality in sample of trauma patients from National Trauma Data Bank (NTDB) with respect to clinical and injury characteristics.

	Died		*P*-value
	No (*N* = 1,180)	Yes (*N* = 558)	
**GCS**			
Severe ≤ 8	201 (18.0%)	124 (22.9%)	0.029
Moderate 9–12	77 (6.9%)	44 (8.1%)	
Mild 13–15	839 (75.1%)	374 (69.0%)	
**SBP**			
≤ 90	141 (12.2%)	95 (17.5%)	0.003
≥ 91	1,012 (87.8%)	448 (82.5%)	
**Transfusion blood (4 h)**			
No	791 (71.1%)	357 (68.3%)	0.247
Yes	322 (28.9%)	166 (31.7%)	
**ISS**			
≤ 15	513 (43.5%)	224 (40.3%)	0.205
≥ 16	666 (56.5%)	332 (59.7%)	
**Trauma type**			
Blunt	969 (82.7%)	476 (85.8%)	< 0.001
Penetrating	167 (14.2%)	48 (8.6%)	
Burn	17 (1.5%)	21 (3.8%)	
Other/unspecified	19 (1.6%)	10 (1.8%)	
**Mechanism of injury**			
Fall	442 (37.8%)	235 (42.9%)	0.016
Firearm	137 (11.7%)	39 (7.1%)	
MVT	422 (36.1%)	200 (36.5%)	
Other	168 (14.4%)	74 (13.5%)	
**Nature of injury**			
Fracture	468 (39.9%)	209 (37.7%)	0.852
Internal organ injury	452 (38.6%)	223 (40.2%)	
Open wound	87 (7.4%)	38 (6.8%)	
Superficial and contusion	64 (5.5%)	34 (6.1%)	
Other	101 (8.6%)	51 (9.2%)	

Table 4.2 summarizes associations of mortality with patient clinical characteristics, treatment parameters and injury characteristics. Mortality was significantly associated with GCS, SBP, trauma type and mechanisms of injury. No statistical significance was reported among blood transfusion, ISS and nature of injury with mortality. Data extracted from the National Trauma Data Bank, 2024 United States.

**TABLE 5:** Crude and adjusted odds ratios of mortality in trauma patients who developed sepsis as hospital complication in trauma centers level II and III compared to trauma center level I.

	Crude			Adjusted		
	OR	95% CI	*P*-value	OR	95% CI	*P*-value
**Trauma designation level (I)**						
II	0.911	0.731–1.136	0.406	0.785	0.592–1.043	0.095
III	1.014	0.673–1.527	0.947	1.038	0.454–2.372	0.930

OR, odds ratio; CI, confidence interval. Multivariable logistic regression demonstrated no significant association between mortality and trauma level designations II and III compared to trauma level I in patients who developed sepsis as hospital complication after adjusting for all possible clinically or statistically significant variables.

## 4 Discussion

The new sepsis definition along with the qSOFA score has allowed earlier sepsis detection and prompt treatment initiation with fluids and antibiotics (25, 26). Our study showed that Level I trauma centers managed most injuries in university hospitals, had higher ICU and OR admissions and reported the highest level of complications including DVT, pressure ulcers, VAP, and unplanned reoperations. This was consistent with previous findings where high complication rates were attributed to more aggressive treatment relative to other trauma center levels (9, 27). In addition, patients treated in a Level I trauma center had higher ISS, lower GCS scores, more blunt trauma from motor vehicle trauma, and were more likely to receive blood transfusions. Most cases presenting to Levels II and III trauma centers were seen at community and non-teaching hospitals, and most Level II admissions were to the ICU or OR, whereas Level III had the highest proportion of general ward admissions. Finally, our study showed that mortality was significantly associated with age, negative alcohol screen, GCS, SBP, trauma type and mechanism of injury, but not with trauma level designation. There was no association between trauma center level and mortality from sepsis in trauma patients once all clinically and statistically significant variables were accounted for (*p* = 0.691).

Studies on patient outcomes presenting with similar injuries across trauma centers of varying levels show conflicting results (11). Several have shown that Level I trauma centers have higher survival rates for patients with severe injuries (ISS > 15 and > 25; head acute injury score ≥ 3) compared to Level II centers (8–14), while Level II centers show no notable survival advantage over lower-level centers, especially in mild or moderate injuries (ISS ≤ 15). A systematic review and meta-analysis by Van Ditshuizen et al. (28) further support this, demonstrating a survival benefit for the severely injured patients (Abbreviated Injury Scale score AIS ≥ 3) presenting to level I trauma center as compared to a level II center. On the other hand, a study by Dekassian et al. (29) showed that in patients presenting with no signs of life from a traumatic injury, higher survival was noted in level II trauma centers compared to level I. According to the authors, this was hypothesized to be related to the increased availability of residency programs in a level I trauma center which was associated with poorer outcomes. Furthermore, higher mortality in older patients and after blunt traumas reported in our study align with existing literature. Two studies reported lower survival rates in elderly trauma patients compared to younger counterparts with similar injuries (30, 31), attributing this to severe preexisting diseases and higher likelihood of developing multiple deleterious complications perpetuating organ dysfunction and accelerated death (30). Additionally, a systematic review by Battle et al concluded that patients over 65 years old with pre-existing cardiorespiratory conditions face higher mortality rates after blunt chest trauma involving three rib fractures (32). In a similar manner to our results, several studies showed no difference in mortality between Level I and Level II trauma centers in settings of blunt trauma, motorcycle crash, and drowning incidents (33–38). The authors of these studies attributed their findings to the maturation of trauma centers, which lead to standardization of care across different levels and resulted in relatively similar outcomes across various traumatic encounters. Furthermore, no survival benefit was observed after trauma in pregnant patients, patients with injuries requiring thoracotomy, and patients after severe head injury treated in different level trauma centers, likely due to effective field triage, ACS management guidelines, and trauma surgeons’ expertise (39–41).

Our study builds upon and differs from previous research in several key ways. To the best of our knowledge, while prior studies extensively examined trauma-related mortality across different trauma center levels, this is the first to explore potential disparities in sepsis-related mortality among the three level trauma centers. Sepsis increases the risk for mortality and leads to worse outcomes in trauma patients even in highest level trauma centers. A study by Chung et al. (42) reported significantly higher mortality rates and longer ICU stays in trauma patients who developed sepsis compared to their non-sepsis counterparts treated at a Level I trauma center, suggesting poorer outcomes in trauma patients who survived the initial period then developed sepsis. Our results have shown that sepsis-related mortality is not influenced by trauma center designation level While trauma management focuses on resuscitating patients safely through the critical hours immediately following trauma, sepsis management on the other hand, has evolved to focus mostly on early recognition and early treatment with fluids and antibiotics (43). Throughout the years, there has been a shift from more aggressive, invasive sepsis management that was proposed by the early goal directed therapy to a strategy of early antibiotics and usual care such as was proposed by the sepsis trilogy trials (44). Therefore, should trauma patients survive the initial critical period and then develop sepsis; it is reasonable to say that if it is rapidly identified and treated with antibiotics, their outcomes are similar regardless of trauma designation level. This highlights that alongside optimizing trauma care, implementing standardized sepsis protocols across all trauma centers is essential to improving trauma patient survival. Early recognition through validated screening tools and timely intervention using protocolized treatments and evidence-based guidelines are key to sepsis care standardization (45). While scoring indices and qSOFA scores remain valuable tools for predicating sepsis and assessing severity, respectively (46, 47), newer advanced machine learning models are also being studied for their potential to provide early sepsis warnings in critical patients. Su et al. (48) investigated predictors of sepsis outcomes in ICU patients using three different machine learning models, all of which outperformed the SOFA score in predictive accuracy. We believe integrating similar models along with standardized protocols into trauma care would enhance early sepsis recognition and support faster intervention, underscoring the need for unified, technology-driven sepsis management across trauma systems.

This retrospective study leveraged a substantial sample size of trauma patients with sepsis from the NTDB—the largest trauma registry in the US—to provide fundamental insights into mortality outcomes across trauma level centers. The registry offers real-world evidence on trauma patient complications, management, and outcomes, reflecting current clinical practices and enhancing the reliability of our findings. Moreover, data from multiple institutions ensures a diverse patient population, thereby improving generalizability across the larger US population. The high quality of NTDB data enables the detection of statistically and clinically significant associations between variables and outcomes. Additionally, insights from NTDB studies help benchmark trauma care quality and identify areas for clinical improvement on a broad scale. Consequently, our findings offer robust and valuable insights into optimizing trauma care and reducing mortality in patients who develop sepsis, regardless of trauma center designation.

However, several limitations are introduced given the retrospective nature of the dataset. First, NTDB excludes patients who are announced dead on scene and not transported to the ED, potentially overestimating survival rates across the different trauma level centers. Second, the majority of eligible patients in the dataset were treated at Level I and Level II trauma centers, resulting in a relatively small sample size for Level III centers that may have compromised precision and reliability of estimates related to Level III trauma care. Moreover, no data is available in the NTDB about sepsis onset timing, time to antibiotic initiation, fluid resuscitation, adherence to sepsis protocols and key biomarkers (lactate, procalcitonin, CRP, caspase-1) which are essential for adequate sepsis management. Also, NTDB has no data on differences in patient acuity. This in turn hinders our understanding of the interplay between the traumatic injury, infections, patients’ sickness status and sepsis, as well as their combined impact on patient outcomes and mortality. Finally, hospitals registered in the NTDB may differ by the quality of the data they report in the dataset even though it is consistently reviewed as part of data and quality assurance (18).

## 5 Conclusion

This study highlights the complex interplay between trauma center designation and the outcomes of trauma patients who developed sepsis as a hospital complication. While Level I trauma centers treated patients with greater injury severity and a higher burden of complications, overall sepsis-related mortality did not differ between trauma levels when adjusted for confounders. These insights underscore the importance of adopting standardized sepsis management protocols across all trauma level centers to optimize trauma care and improve patient survival outcomes.

## Data Availability

The raw data supporting the conclusions of this article will be made available by the authors, without undue reservation.
